# Invasive fungal sinusitis in patients with hematological malignancy: 15 years experience in a single university hospital in Taiwan

**DOI:** 10.1186/1471-2334-11-250

**Published:** 2011-09-22

**Authors:** Chien-Yuan Chen, Wang-Huei Sheng, Aristine Cheng, Yee-Chun Chen, Woei Tsay, Jih-Luh Tang, Shang-Yi Huang, Shan-Chwen Chang, Hwei-Fang Tien

**Affiliations:** 1 Departments of Internal Medicine, Division of Hematology, National Taiwan University Hospital, (No. 7 Chung-Shan South Road), Taipei, (100), Taiwan; 2 Departments of Internal Medicine, Division of Infectious Disease, National Taiwan University Hospital, (No. 7 Chung-Shan South Road), Taipei, (100), Taiwan

**Keywords:** Invasive fungal sinusitis (IFS), hematological disease, Aspergillus galactomanan

## Abstract

**Background:**

Risk factors and outcomes in hematological patients who acquire invasive fungal sinusitis (IFS) are infrequently reported in the modern medical era.

**Method:**

A retrospective study of hospitalized patients with hematological disease was conducted at National Taiwan University Hospital between January 1995 and December 2009.

**Results:**

Clinical characteristics and outcomes with their associated radiographic and microbiological findings were analyzed. Forty-six patients with IFS and 64 patients with chronic non-invasive sinusitis were enrolled as comparsion. IFS developed more commonly in patients with acute myeloid leukemia (AML) and with prolonged neutropenia (absolute neutrophil count less than 500/mm^3 ^for more than 10 days) (*p *< 0.001). *Aspergillus flavus *was the most common pathogen isolated (44%). Serum *Aspergillus *galactomannan antigen was elevated in seven of eleven patients (64%) with IFS caused by aspergillosis but negative for all three patients with mucormycosis. Bony erosion and extra-sinus infiltration was found in 15 of 46 (33%) patients on imaging. Overall, 19 of 46 patients (41.3%) died within 6 weeks. Patients with disease subtype of AML (p = 0.044; Odds Ratio [OR], 5.84; 95% confidence interval [95% CI], 1.02-30.56) and refractory leukemia status (p = 0.05; OR, 4.27; 95% CI, 1.003-18.15) had worse prognosis. Multivariate analysis identified surgical debridement as an independent good prognostic factor (p = 0.047) in patients with IFS.

**Conclusions:**

Patients of AML with prolonged neutropenia (> 10 days) had significantly higher risk of IFS. Early introduction of anti-fungal agent and aggressive surgical debridement potentially decrease morbidity and mortality in high risk patients with IFS.

## Background

Chemotherapy is still the current standard treatment for hematological malignancy and febrile neutropenia is a common complication of chemotherapy in most patients [1]. If fever persists 72-96 hours after treatment with broad-spectrum antibacterial antibiotics during neutropenia, invasive fungal infection may be suspected and treated empirically with antifungal agents [1-3]. However, since the culture methods are insensitive and radiologic findings are nonspecific, the diagnosis of invasive fungal infection remains a challenge [4]. An international consensus on the diagnosis of opportunistic invasive fungal infections in immunocompromised patients with cancer and hematopoietic stem cell transplants was established by the Invasive Fungal Infections Cooperative Group of the European Organization for Research and Treatment of Cancer (EORTC) and the Mycoses Study Group of the National Institute of Allergy and Infectious Diseases (MSG-NIAID) [5,6]. Delay in the treatment of invasive fungal infection during neutropenia causes high mortality in patients with transplants and hematological malignancy [4,5,7]. The uncertainty in disease diagnosis results in under- or over-treatment of invasive fungal infection. Despite the availability of several new antifungal agents, including triazoles and echinocandins, the effectiveness of antifungal therapy remains uncertain and the effectiveness of neutrophil recovery may not be sufficient if the recovering neutrophils are dysfunctional [8].

Invasive fungal sinusitis (IFS) is a rare disease largely attributable to *Aspergillus *and *Mucor *in patients with stem cell transplants and hematological disease [9]. Though the mortality of IFS in immunocompromised patients ranges from 50% to 80% [7,9,10], early physical findings are non-specific and ambiguous (i.e., nasal obstruction, purulent discharge, and epistaxis). Water's view plain radiographs do not distinguish invasive fungal sinusitis from chronic allergic sinusitis. Bony erosion and tissue destruction is often found only in the advanced stage by computed tomography [11,12]. Recent introduction of serial *Aspergillus *galactomannan antigen test may provide early evidence of IFS. In this study, clinical characteristics were retrospectively reviewed and their correlation with outcome was evaluated to identify prognostic factors in patients with hematological disorders and IFS.

## Methods

### Patients

National Taiwan University Hospital is a 2200-bed teaching hospital that provides both primary and tertiary care in metropolitan Taipei. More than 600 admissions annually comprise adult patients with hematological malignancies for in-patient chemotherapy. Lymphoma, acute myeloid leukemia and myeloma were the three most common hematological diagnoses [13].

The search terms "sinusitis" and "sinonasal infection" were used to identify reports in the discharge summary database of all patients admitted to the Department of Internal Medicine between January 1995 and December 2009. Patients with hematopoietic stem cell transplants, hematological disease (including severe aplastic anemia, pure red cell aplasia, and hematological malignancy), and sinusitis diagnosed during their hospital stay were enrolled in this study. Patients with hematological disorders and chronic non-invasive sinusitis admitted in the same period of time cohort were enrolled as controls. Demographic features, hematological disease status, underlying medical diseases, chemotherapy regimen before diagnosis of fungal sinusitis, evidence (clinical, microbiological, pathological, and radiological) showing fungal sinusitis, antifungal therapy, and outcome were collected and analyzed retrospectively. This research conformed to the Helsinki Declaration and local legislation, and approved by the local ethics committee.

### Diagnosis of Sinusitis

During the study period, patients with hematological malignancy developed sinusitis were routinely to take sinus X-ray and otolaryngist was consulted for focal evaluation and tissue culture. CT and MRI sinus study, surgical biopsy and debridement were performed according to clinicians' decision.

### Diagnosis, Prophylaxis and Treatment of invasive fungal sinusitis

Physicians clinically diagnosed IFS in all these patients according to EORTC/MSG-NIAID consensus criteria in 2008 [5,6]. The host factors included prolonged neutropenia (< 500 neutrophils/mm^3 ^for > 10 days) temporally related to the onset of fungal disease, receipt of an allogeneic stem cell transplant, prolonged use of corticosteroids, immunosuppressive agents, or nucleoside analogues during the past 90 days. The clinical criteria included imaging showing sinusitis plus at least one of the following three signs: acute localized pain, nasal ulcer with black eschar, extension from the paranasal sinus across the bony barrier. The microbiological criteria included culture or isolation of fungus from surgical material or sinus aspirate samples, and detection of *Aspergillus *galactomannan antigens in serum. In our institution, an optical density (OD) index of 0.5 or higher is considered positive for aspergillus galactomannan test.

No routine anti-fungal prophylaxis was prescribed in hematological patients receiving standard chemotherapy. Fluconazole was used as antifungal prophylactic agent in the recipients of stem cell transplantation. Timing and choice of pre-emptive anti-fungal therapy was based on clinical judgment according to the presence of any of the following high risk factors: local symptoms and signs of sinusitis, elevated serum *Aspergillus *galactomannan antigen, or abnormal CT imaging. The maintenance dose of each antifungal agent is as following: amphotericin B 1-1.5 mg/kg/day, amphotericin B liposomal 3-5 mg/kg/day, capsofungin acetate 50 mg/day, and voriconazole 400 mg/day.

### Proven, probable, and possible invasive fungal sinusitis

Proven, probable, and possible IFS were defined mainly according to the EORTC/MSG-NIAID criteria [5,6]. Proven IFS was defined by the presence of fungi associated tissue damage on histopathologic examination of a biopsy specimen; or positive culture result, consistent with infection, from a sample obtained aseptically from a clinically or radiologically abnormal site. Probable IFS was defined by the presence of at least one host factor criterion, one microbiological criterion, and one clinical criterion. Possible IFS was defined by the presence of at least one host factor criterion and one clinical criterion.

### Survival and Mortality

The survival is defined from diagnosis of invasive fungal sinusitis to patients died. Attributable mortality to invasive fungal sinusitis is defined if patient died within 6 weeks, and there were no other attributable cause of death.

### Statistics

Comparisons were made using the chi-square test. Predictors of survival were identified using a Cox regression model. Factors which p level is below 0.2 in the univariate analysis were selected for multivariate Cox regression analysis. All statistical analyses were performed using SPSS 13.0 for Windows (SPSS, Chicago, IL, USA). A p value less than 0.05 was considered statistically significant.

## Results

### Clinical characteristics of patients with IFS

The diagnosis of invasive fungal sinusitis was made in 46 patients with hematological disorders, and 64 patients with hematological disorders and chronic non-invasive sinusitis admitted in the same period of time cohort were enrolled as controls. There was no outbreak of invasive fungal sinusitis detected in this study. The overall incidence of IFS is 1.77 per 1000 patients. The incidence and trend of IFS in adult hematological patients was shown in Table 1. There were trends of decreasing incidence and mortality during the study period, although these did not attain statistically significance (p = 0.199 and 0.223).

**Table 1:** Comparison of three different periods of incidence and mortality of patients with hematological disorders and invasive fungal sinusitis (IFS) by χ^2 ^trend analysis (p = 0.199).

	Year			
		
	1995-1999	2000-2004	2005-2009	*p*
Case number	568	674	1355	
Patient number of IFS	13	11	22	
Incidence of IFS	2.29%	1.63%	1.62%	0.199
Patient number of mortality	6	6	7	
Mortality rate of IFS	46.2%	54.5%	31.8%	0.223

IFS was proven in 25 patients (54%), probable in 5 patients (11%), and possible in 16 patients (35%). Their clinical characteristics are shown in Table 2. Compared with chronic non-specific sinusitis, IFS was more common in patients with disease subtype of acute myeloid leukemia (AML) than in patients with other types of hematological malignancy (p < 0.001) and more common in patients with absolute neutrophil count less than 500/mm^3 ^for ≥10 days. The occurrence of IFS was not affected by underlying diabetes mellitus, hematological disease status, chemotherapy intensity, and allogeneic stem cell transplantation.

**Table 2:** Clinical characteristics of patients with invasive fungal sinusitis.

		Sinusitis			EORTC/MSG-NIAID IFS				
						
	Total (n = 110)	IFS (n = 46)	Non-IFS (n = 64)	*P*	Proven (n = 25)	Probable (n = 5)	Possible (n = 16)	Non-IFS (n = 64)	*P*
Age									
< 60 years	86	34	52	0.48	19	5	10	52	0.25
≥ 60 years	24	12	12		6	0	6	12	
Gender									
Male	66	27	39	0.85	15	3	9	39	0.99
Female	44	19	25		10	2	7	25	
Hematological diagnosis				< 0.001					0.004
Acute myeloid leukemia	56	33	23		16	4	13	23	
Acute lymphoblastic leukemia	16	6	10		6	0	0	10	
Lymphoma/myeloma	20	0	20		0	0	0	20	
SAA/MDS	9	4	5		2	0	2	5	
Others*	9	3	6		1	1	1	6	
Disease status				0.24					0.38
Fresh diagnosis/remission	44	15	29		10	1	4	29	
Refractory/relapsed	66	31	35		15	4	12	35	
Underlying diabetes mellitus				0.45					0.68
Yes	7	4	3		2	1	1	3	
No	103	42	61		22	5	15	61	
Chemotherapy intensity**				0.21					0.045
Supportive treatment	34	11	23		3	4	4	23	
Low dose chemotherapy	13	8	5		3	1	4	5	
Standard dose chemotherapy	47	22	25		16	0	6	25	
High dose chemotherapy	16	5	11		3	0	2	11	
Absolute neutrophil count				< 0.001					< 0.001
< 500 mm^3 ^< 10 days	55	7	48		2	1	4	48	
< 500 mm^3 ^≥ 10 days	55	39	16		23	4	12	16	
Allogeneic transplantation				0.07					0.013
Yes	24	6	18		2	3	1	18	
No	86	40	46		23	2	15	46	
FESS debridement				0.001					< 0.001
Yes	18	14	4		12	0	2	4	
No	92	36	56		20	1	15	56	
Pre-emptive antifungal agent				< 0.001					< 0.001
Amphotericin B/ambisome		28	12		14	4	10	12	
Caspofungin		0	1		0	0	0	1	
Voriconazole		2	0		2	0	0	0	
Amphotericin B and caspofungin		4	0		3	0	1	0	
Amphotericin B and voriconazole		9	1		6	0	3	1	
Outcome (6 weeks survival)				0.001					0.001
Death	27	19	8		7	3	9 1110	8	
Alive	83	27	56		18	2	7	56	

Abbreviation: IFS, invasive fungal sinusitis; SAA, severe aplastic anemia; MDS, myelodysplastic syndrome, FESS: functional endoscopic sinus surgery.

*Others including: chronic myeloid leukemia (2), chronic lymphocytic leukemia (3), T-large granular lymphocyte leukemia (1), myelofibrosis (2), pure red cell aplasia status post transplantation (1)

**High dose chemotherapy includes high dose cytarabine chemotherapy and conditioning therapy before myeloablative transplantation.

### Microbiological spectrum

Among the 25 patients with proven invasive fungal sinusitis, fifteen patients had positive biopsy showed hyphae with Giemsa stain or PAS stain, and 14 patients showed positive fungal cultures. The fungal isolates from 25 patients with proven invasive fungal sinusitis included: *Aspergillus flavus *(12), *Aspergillus fumigatus *(2), *Aspergillus vesicolor *(1), *Aspergillus sybowii *(1), unidentified Aspergillus species (1), *Mucor *(4), *Phaeohyphomyces *(1), *Fusarium *(1), *Penicillium *(1), and unidentified fungal species (1).

### *Aspergillus *galactomannan antigen test

Sixteen patients during 2005 to 2009 had serial follow-up of *Aspergillus *galactomannan antigen. The mean value (+/- SD) of *Aspergillus *galactomannan antigen test is 2.32 (+/- 1.86). The mean value (standard deviation) of Aspergillus galactomannan antigen in the seven patients with positive results was 2.32 (1.86), compared with those with negative results 0.13 (0.07). The p value (0.048) is below 0.05. *Aspergillus *galactomannan antigen was not elevated in three patients with mucormycosis sinusitis. *Aspergillus *galactomannan antigen was elevated in seven of eleven patients with *aspergillus *sinusitis. One patient with *phaeohyphomycosis*-related sinusitis and one patient with sinusitis caused by *Fusarium *and *Penicillium marneffei *also showed elevated *aspergillus *galactomannan antigen. The sensitivity of serum galactomannan antigen testing was 64% and the specificity was 60% for the detection of invasive *aspergillus *sinusitis. (Table 3)

**Table 3:** Aspergillus galactomannan test for detect invasive aspergillosis

	Patients with Aspergillus sinusitis (n = 11)	Patients with non-Aspergillus sinusitis* (n = 5)
Aspergillus galactomannan test		
Positive test	7	2
Negative test	4	3

*Patients with non-Aspergillus sinusitis: mucormycosis, (3 patients); phaeohyphomycosis, (1); fusariosis/penicilliosis (1)

### Radiological manifestations

Sinus imaging by computed tomography (CT) or magnetic resonance (MRI) were performed in 44 (96%) of patients with IFS and 37 (59%) of 63 patients with chronic non-specific sinusitis. Evidence of extra-sinus tissue involvement and/or bony destruction were detected in 15 (33%) of patients with IFS. In addition, brain abscess complicating IFS was found in two patients and cavernous sinus thrombosis in one patient.

### Treatment and outcome

Pre-emptive antifungal treatment was given in 43 (94%) of 46 patients with IFS. Fourteen of sixty-four patients with non-IFS received pre-emptive antifungal therapy initially, and these agents were discontinued after excluding the diagnosis of IFS. The overall mortality rate of patients with IFS was 41% (Table 2). The attributable mortality of patients with AML was also much higher than non-AML patients. All the patients died rapidly due to IFS (median survival, 15 days; 25^th ^and 75^th ^percentile, 10 and 34 days, respectively). Six-week survival was not significantly different between patients who had received antifungal combination therapy (amphotericin B plus capsofungin and amphotericin B plus voriconazole vs amphotericin B) compared to those who had received monotherapy (p = 0.74). All four patients with *mucormycoses *had received high dose antifungal therapy and aggressive surgical debridement, but three died within 6 weeks. Twelve patients with proven invasive fungal sinusitis and two patients with possible invasive fungal sinusitis underwent functional endoscopic sinus surgery for debridement. The median interval between diagnosis and surgical debridement is 11.5 days (range 1-38 days). Nineteen of 46 patients (41%) with IFS and 8 of 64 patients (13%) with non-invasive sinusitis died within 6 weeks after diagnosis of sinusitis. The 6-week mortality rate was significantly higher in patients with IFS than those with non-invasive sinusitis (p = 0.001). Univariate analysis of 6-week outcomes in patients with hematological disorders and invasive fungal sinusitis is summarized in Table 4. The acute myeloid leukemia disease subtype (p = 0.044) and refractory disease status were associated with worse outcomes (p = 0.05). The intensity of chemotherapy and allogeneic stem cell transplantation were not independent prognostic factors of invasive fungal infection in this study. The median interval between neutropenia and diagnosis of IFS was not significantly different between non-survivors and survivors (median 19 days vs 21 days, *p *= 0.677). Multivariate Cox regression analysis revealed functional endoscopic sinus surgery for debridement as the only independent prognostic factor for survival (p = 0.047).

**Table 4:** Univariate analysis outcome in patients with hematological disorders.

	IFS (n = 46)		*P*	Odds Ratio	95%CI
				
	Alive (n = 27)	Dead (n = 19)			
Age			0.51		
≥ 60 years	6	6			
< 60 years	21	13			
Gender			0.55		
Male	17	10			
Female	10	9			
Hematological diseases			0.04	5.84	1.02-30.56
AML	16	17			
Non-AML*	11	2			
Disease status			0.05	4.27	1.003-18.15
Fresh diagnosis/remission	12	3			
Relapse/refractory	15	16			
Underlying diabetes mellitus			> 0.99		
Yes	2	2			
No	25	17			
Chemotherapy intensity			0.25		
Supportive care	5	6			
Low dose chemotherapy	3	5			
Standard dose chemotherapy	16	6			
High dose chemotherapy	3	2			
Absolute neutrophil count			0.68		
< 500 mm^3 ^< 10 days	5	2			
< 500 mm^3^≥10 days	22	17			
Allogeneic transplantation			0.68		
Yes	3	3			
No	24	16			
FESS debridement			0.10	0.27	0.064-1.17
Yes	11	3			
No	16	16			
Fungus type			0.21		
Aspergillosis	12	5			
Mucormycosis	2	2			
Others**	2	2			
Unknown	10	12			
Invasive image pattern***			0.34		
Yes	7	8			
No	20	11			
Pre-emptive antifungal agent			0.29		
Amphotericin B/ambisome	15	13			
Caspofungin	0	0			
Voriconazole	2	0			
Amphotericin B and caspofungin	1	3			
Amphotericin B and voriconazole	7	4			

Abbreviation: AML, acute myeloid leukemia; FESS: functional endoscopic sinus surgery.

*Non -AML: including acute lymphoblastic leukemia 6 cases, severe aplastic anemia/myelodysplastic syndrome, 2 cases, other: CLL, 2 cases, CML 1 case

**. Others: Phaeohyphomycosis, *Fusarium *mycosis, *Penicillium *mycosis, and unidentified mycosis

***. Invasive image pattern indicates brain abscess, invasion of tissue.

## Discussion

In this large retrospective study, we estimated IFS occurred in 1.77% of hospitalized patients with hematological disorders. IFS caused significantly higher mortality in AML patients with prolonged neutropenia (> 10 days). We identified surgical debridement is an independent factor associated with good outcome.

IFS developed more frequently in patients with AML, myelodysplastic syndrome, and aplastic anemia, but not at all in patients with lymphoma/myeloma. In the literature review, most patients with lymphoma who developed IFS are recipients of myeloablative allogeneic stem cell transplants [14-18]. Compared with other subtypes of hematological malignancy, patients with AML had significantly higher risk of IFS. The risk of developing IFS in AML relates to neutropenia (p < 0.001) and less to the intensity of chemotherapy regimens. The mortality of patients with AML was also much greater than non-AML patients.

Prolonged neutropenia in patients with myeloid malignancies may contribute to underlying disease, intensity and dosage of chemotherapy, colony-stimulating factor, and concurrent medication. Invasive mold infection often occurs when a large burden of spores from an environmental source is deposited on mucosal membranes lacking an effective phagocytic host defense [19]. Using cytokine growth factors to decrease the period of chemotherapy-associated neutropenia and using laminar air flow rooms for protection against IFS [20] may reduce the risk of IFS after allogeneic stem cell transplantation [21]. In this study, we found a lower incidence and mortality of IFS in 2005-2009 than in 1994-1999.

The clinical mycological spectrum of IFS is limited in patients with stem cell transplants and hematological disease [14-18]. *Aspergillus *and *Mucor *are the main mold found in biopsy, however, the prevalence is highly variable in different geographic regions [14-18,22,23]. In our study, *Aspergillus flavus *(44%) was the most common isolate. *Aspergillus flavus*, with its unique ability to survive at higher temperatures, is the predominant pathogen in countries, including most of the Middle East, Africa, and Southeast Asia [24,25]. Rare IFS in Asia and Africa were reported, the clinical response varies differently with fungal subtypes, and further epidemiology study should be investigated. *Mucormycosis *is an emerging cause of IFS with a rapid fatal course in patients with hematological disorders [26,27]. In our study, all four patients with *mucormycoses *had received high dose antifungal therapy and aggressive surgical debridement, but three died within 6 weeks (mortality, 75%). Effective treatment for *Mucormycosis *should be investigated.

The symptoms and signs of paranasal sinusitis (such as nasal discharge, stuffiness, epistaxis, periorbital swelling, and maxillary tenderness) are nonspecific for IFS. Symptoms and signs such as nose ulceration, eschar of the nasal mucosa, black necrotic lesions, and perforation of the hard palate are more specific, but these findings are present only at an advanced stage. The use of CT and MRI in the diagnosis of invasive fungal sinusitis has been reported [12]. Diagnostic radiological evidence of invasive fungal sinusitis includes erosion of sinus walls, extension of infection to neighboring structures, and extensive skull base destruction. However, most patients do not have classic findings in the early phase of invasive fungal sinusitis. Earlier diagnosis by using serial Aspergillus galactomannan antigen test in the modern medical era to detect IFS, may lead to early introduce anti-fungal agent and surgical debridement, and potentially decreased morbidity and mortality in high risk patients.

There are some limitations in this study. First, this is a retrospective time cohort study in a single university institution in Taiwan. Therefore, information of laboratory results, such as *Aspergillus *galactomannan antigen test, might be limited. Second, surgical approach of sinusitis were performed according to clinicians' decision, thus, the etiology of control patients were not available. Third, as IFS is not a common disease in patients with heamatological disorder, the case numbers is limited.

In conclusion, outcome of IFS is usually very poor [11,28]. In this study, functional endoscopic sinus surgery for debridement was the only indicator of better prognosis in multivariate analysis. This result confirms previous findings indicating that sinus surgery improves outcome [28,29]. Aggressive surgical debridement combined with antifungal therapy, should be emphasized in leukemic patients in spite of their prolonged neutropenia and bleeding tendencies. Whether the intensity of chemotherapy and allogeneic stem cell transplantation were the independent prognosis of invasive fungal infection need further investigation.

## Competing interests

The authors declare that they have no competing interests.

## Authors' contributions

CYC, WHS, and AC designed, conducted the study and wrote manuscript. YCC, WT, JLT, SYH, SCC, HFT recruit patients and provide patients care. All authors read and approved the final manuscript.

## Pre-publication history

The pre-publication history for this paper can be accessed here:

http://www.biomedcentral.com/1471-2334/11/250/prepub
